# Loss of Medicaid Coverage During the Renewal Process

**DOI:** 10.1001/jamahealthforum.2024.0839

**Published:** 2024-05-03

**Authors:** Laura Dague, Rebecca Myerson

**Affiliations:** 1 Texas A&M University, College Station; 2 University of Wisconsin–Madison, Madison

## Abstract

**Importance:**

Medicaid beneficiaries must periodically redemonstrate their eligibility in a process that is called renewal, redetermination, or recertification. The number and characteristics of people who lose Medicaid coverage due to renewal requirements are not known.

**Objective:**

To measure the proportion of people who lose Medicaid coverage at the renewal deadline, overall and by enrollee characteristics, and time until regaining Medicaid coverage among those losing coverage at the deadline.

**Design, Setting, and Participants:**

This cohort study tracked the duration of Medicaid enrollment among Wisconsin Medicaid enrollees with a 12-month renewal deadline. Data were collected for all nonelderly (aged <65 years) new enrollees from January 2016 through January 2018, except those enrolled due to disability or pregnancy. Individuals were followed through January 2020 to provide at least 24 months of data on each enrollment spell. Data were analyzed from August 2023 to February 2024.

**Main Outcomes and Measures:**

The primary outcome was coverage loss during the renewal process, defined as a loss in Medicaid coverage from month 12 to month 13 for people who were still enrolled at the start of month 12. Secondary outcomes included coverage loss prior to the renewal deadline and the duration of the gap in Medicaid coverage among those who lost coverage during the renewal process.

**Results:**

The study sample included 684 245 Medicaid enrollment spells across 586 044 people (51% female and 47% children 18 years or younger). Among enrollees, 20% lost Medicaid coverage at the renewal deadline. Of those who lost coverage, 37% regained Medicaid coverage within 6 months, and an additional 10% regained coverage within 12 months. Children younger than 12 years and people with more Medicaid-covered health care (top quartile of Medicaid-covered health care costs during the first 6 months of enrollment) were less likely than other groups to lose coverage during the renewal process (15% and 6% lost coverage at renewal, respectively) and more likely to regain Medicaid quickly. Personal characteristics such as gender and race and ethnicity remained associated with the risk of losing Medicaid at the renewal deadline after adjustment for baseline household income, enrollment group, and past use of Medicaid services.

**Conclusions and Relevance:**

In this cohort study, the risk of coverage loss during the Medicaid renewal process was associated with age, past use of care, and other personal characteristics. These findings shed light on how renewal requirements shape access to Medicaid.

## Key Points

**Question:**

Who loses Medicaid coverage during the annual renewal process?

**Findings:**

In this cohort study of 684 245 Medicaid enrollment spells across 586 044 people in Wisconsin, 1 in 5 beneficiaries lost coverage at their 12-month renewal deadline, though this risk was lower for children younger than 12 years and people who had used more Medicaid-covered care. Personal characteristics such as gender and race and ethnicity were associated with risk of losing Medicaid at the renewal deadline after adjustment for baseline household income, enrollment group, and past use of Medicaid services.

**Meaning:**

The risk of coverage loss during the Medicaid renewal process is associated with age, past use of care, and other personal characteristics.

## Teaser

This cohort study measures the proportion of people who lose Medicaid coverage at the renewal deadline, overall and by enrollee characteristics, and time until regaining Medicaid coverage among those losing coverage at the deadline.

## Introduction

Each year, millions of individuals with low income must redemonstrate their eligibility for Medicaid to avoid losing benefits in a process that is called recertification, redetermination, or renewal.^1,2^ While the goal of this requirement is to prevent benefit receipt among people who are ineligible, people who are eligible for benefits also lose coverage if they do not complete the required processes.^3,4,5,6,7,8,9^ Policymakers have raised concerns that administrative difficulties with renewal of coverage may prevent fair and equal access to benefits among all who are eligible.^10,11^

Losing Medicaid coverage can result in a variety of adverse effects. Historically, half of people disenrolled from Medicaid for any reason have not transitioned to another identified insurance (ie, became uninsured).^12,13,14,15,16,17^ Losing Medicaid coverage reduces access to care and raises the risk of impoverishment due to medical debt.^18,19,20,21^ Identifying factors associated with Medicaid coverage loss can inform efforts to help eligible beneficiaries keep their coverage; however, little is known about who typically loses eligibility for Medicaid during renewal. This issue attracted public attention in April 2023 when Medicaid reinstated renewal requirements due to the elimination, or “unwinding,” of the temporary suspension of renewal requirements due to the COVID-19 pandemic, causing millions of people to lose Medicaid coverage.^2,22,23,24,25^ While states are required to publish statistics on how many people lose Medicaid during unwinding, these numbers lack historical comparisons. To inform policy discussions on how frequently Medicaid renewal should be required under “typical” conditions,^26,27,28^ data are needed on who loses coverage during renewal.

This cohort study measures losses of Medicaid coverage during annual renewal processes, including both potentially avoidable losses due to administrative requirements and those due to lack of eligibility. We used administrative data on the universe of nonelderly, nondisabled Wisconsin Medicaid (BadgerCare Plus) members before renewal requirements were suspended due to the COVID-19 public health emergency. The findings will inform policy by identifying groups of people with low rates of renewal who may benefit from targeted outreach and assistance, and by providing a historical benchmark to facilitate interpretation of data from the unwinding process.

## Methods

### Study Setting

We used data from Wisconsin. As in most states, beneficiaries must typically renew their eligibility for Medicaid 12 months after coverage begins (an exception is pregnant individuals, whose renewal date is 2 calendar months after the date their pregnancy ends). Early in their 11th month of Medicaid enrollment, beneficiaries are mailed notices stating that some or all of their benefits will end if renewal is not completed by the end of the following month.^29^ Renewals can be completed in person or via telephone, mail, or online. Coverage ceases if the beneficiary does not complete renewal by the deadline. Late renewals are possible for up to 90 days after the deadline; beneficiaries determined eligible during this period may have their coverage reinstated back to the termination date. People who lose Medicaid may reapply at any time. Thus, eligible people who lose Medicaid coverage may regain it quickly (sometimes called “churning”), whereas people who lose coverage due to ineligibility may remain without Medicaid for a longer period.

This observational cohort study followed the Strengthening the Reporting of Observational Studies in Epidemiology (STROBE) reporting guidelines. The institutional review board at Texas A&M University determined that the analysis did not constitute human subject research and did not require further review.

### Data and Variables

The analysis used administrative data from Wisconsin, including Medicaid enrollment, claims, and encounter data. The study sample included 751 110 new enrollment spells for nonelderly (aged <65 years), nondisabled individuals in Wisconsin Medicaid at any point from January 2016 through January 2018. Individuals were followed up through January 2020 to provide at least 24 months of data prior to the implementation of the Medicaid continuous coverage policy. Data were analyzed from August 2023 to February 2024. A new spell was defined as enrollment following a Medicaid coverage gap of 1 or more months, which would begin a new 12-month renewal period. We excluded spells that began because of pregnancy or foster care–related Medicaid eligibility (n = 39 199) because these renewals follow a different timeline, for those whose age did not match their eligibility category or who were missing eligibility information (n = 54), and for people aged 17 to 18 years or 63 to 64 years at the start of the spell (n = 27 612) because they might lose Medicaid for age-related reasons. Thus, the sample included all Medicaid enrollment spells that began because of income-related eligibility (ie, children, parents/caretakers, childless adults), with 684 245 spells across 586 044 people.

The primary outcome was Medicaid coverage loss during the renewal process, defined as a loss in Medicaid coverage from month 12 to 13 for people still enrolled at the start of month 12. Secondary outcomes included Medicaid coverage loss prior to renewal (ie, spells ending before month 12) and the duration of the gap in Medicaid coverage among those who lost coverage during the renewal process (<6 months, 6-11 months, or ≥12 months). Coverage loss prior to 12 months can occur for various reasons, including loss of eligibility due to a change in income or family circumstances reported to the state, moving out of state, aging out of benefit eligibility, death, or by beneficiary request.

Independent variables were categorized into 2 groups: (1) factors associated with eligibility for and use of Medicaid-covered health care, and (2) demographic factors associated with equity in access to Medicaid. The first set of factors included eligibility group at the start of the enrollment spell (childless adults, parent/caretaker, or child), household income at initial enrollment (<25% of federal poverty level [FPL], 25%-75% of FPL, >75% of FPL, or missing), and health care costs paid by Medicaid during the first 6 months of enrollment (no costs vs first, second, third, or fourth quartile of positive costs in the study sample). The second set of factors included age (<30 years, 30-49 years, or ≥50 years), gender (male or female), education level (<high school, high school, or >high school); race and ethnicity (American Indian or Alaska Native, non-Hispanic; Asian or Pacific Islander, non-Hispanic; Black, non-Hispanic; Hispanic; multiracial; White, non-Hispanic; and unknown), urbanicity (metro or nonmetro area), US citizenship, and tribal membership. Gender and race and ethnicity were generally self-identified but were occasionally reported by caseworkers. Missing data were grouped in a separate category when applicable.

### Statistical Analysis

We used a nonparametric Kaplan-Meier analysis to visually compare rates of coverage loss at the renewal deadline (month 12) vs other months. We calculated rates of Medicaid coverage loss before and at the renewal deadline and duration of coverage loss among those who lost coverage at the renewal deadline, both overall and by enrollee characteristics. We also tested whether there was a statistically significant difference in coverage outcomes by enrollee characteristics using χ^2^ tests.

Next, we used logistic regression to identify factors associated with coverage loss during renewal. The first regression model tested whether factors associated with eligibility for and use of Medicaid-covered health care were associated with coverage loss during the renewal process using an *F* test to assess the joint significance of these variables. The second regression model additionally included demographic characteristics. We tested whether demographic factors remained associated with coverage loss after adjustment for factors associated with eligibility for and use of Medicaid services by using an *F* test to assess the joint significance of demographic variables. We reported average marginal effects and robust standard errors for all logistic models.

Analyses were performed in Stata, version 18 (StataCorp). Two-sided hypothesis tests were conducted with *P* *<* .05 as the threshold for statistical significance.

## Results

The study sample included 684 245 Medicaid enrollment spells from 586 044 people; 51% of sample members were female, and 47% were children 18 years or younger. Table 1 summarizes the characteristics of people in the study sample, both overall and by major eligibility group (childless adult, parent/caretaker, or child). Most sample members lived in metro areas (71%), and 47% had an income less than 25% of FPL. Among sample members, 2% identified as American Indian or Alaska Native, 4% as Asian or Pacific Islander, 19% as Black, 12% as Hispanic, 4% as multiracial, and 51% as White, with 9% unknown.

**Table 1.** Characteristics of People in the Study Sample by Eligibility Category

Characteristic	No. (%)			
	Childless adult	Parent/caretaker	Child^a^	Total
No. of enrollees	175 394 (29.9)	134 324 (22.9)	276 326 (47.2)	586 044 (100.0)
Age, y				
<30	74 255 (42.3)	48 414 (36.0)	276 326 (100.0)	398 995 (68.1)
30-49	61 730 (35.2)	77 798 (57.9)	0	139 528 (23.8)
≥50	39 409 (22.5)	8112 (6.0)	0	47 521 (8.1)
Gender^b^				
Female	70 656 (40.3)	90 034 (67.0)	135 629 (49.1)	296 319 (50.6)
Male	104 738 (59.7)	44 290 (33.0)	140 697 (50.9)	289 725 (49.4)
Education level				
<High school or missing	127 002 (72.4)	79 820 (59.4)	275 882 (99.8)	482 704 (82.4)
High school degree or equivalent	45 627 (26.0)	49 822 (37.1)	415 (0.2)	95 864 (16.4)
>High school	2765 (1.6)	4682 (3.5)	29 (0.0)	7476 (1.3)
Race and ethnicity^b^				
American Indian or Alaska Native, non-Hispanic	3621 (2.1)	2961 (2.2)	5038 (1.8)	11 620 (2.0)
Asian or Pacific Islander, non-Hispanic	4239 (2.4)	5835 (4.3)	10 859 (3.9)	20 933 (3.6)
Black, non-Hispanic	41 934 (23.9)	26 430 (19.7)	43 052 (15.6)	111 416 (19.0)
Hispanic	13 731 (7.8)	14 459 (10.8)	43 077 (15.6)	71 267 (12.2)
Multiracial	4172 (2.4)	3234 (2.4)	15 621 (5.7)	23 027 (3.9)
White, non-Hispanic	100 735 (57.4)	77 869 (58.0)	118 662 (42.9)	297 266 (50.7)
Unknown	6962 (4.0)	3536 (2.6)	40 017 (14.5)	50 515 (8.6)
Urbanicity				
Metro area	129 037 (73.6)	93 514 (69.6)	195 613 (70.8)	418 164 (71.4)
Nonmetro area	35 241 (20.1)	34 335 (25.6)	66 603 (24.1)	136 179 (23.2)
Missing	11 116 (6.3)	6475 (4.8)	14 110 (5.1)	31 701 (5.4)
Income as % of federal poverty level at enrollment				
<25%	121 560 (69.3)	60 168 (44.8)	94 718 (34.3)	276 446 (47.2)
25%-75%	27 815 (15.9)	37 054 (27.6)	52 678 (19.1)	117 547 (20.1)
>75%	21 807 (12.4)	35 442 (26.4)	123 608 (44.7)	180 857 (30.9)
Missing	4212 (2.4)	1660 (1.2)	5322 (1.9)	11 194 (1.9)
US citizenship				
Noncitizen	3303 (1.9)	5313 (4.0)	3661 (1.3)	12 277 (2.1)
Citizen	172 091 (98.1)	129 011 (96.0)	272 665 (98.7)	573 767 (97.9)
Tribal membership				
Not a tribe member	202 013 (97.8)	156 399 (97.4)	335 931 (97.5)	694 343 (97.5)
Tribe member	4557 (2.2)	4173 (2.6)	8784 (2.5)	17 514 (2.5)
Health care costs paid during first 6 mo of Medicaid enrollment				
None	103 530 (59.0)	65 191 (48.5)	108 392 (39.2)	277 113 (47.3)
First quartile	13 144 (7.5)	12 816 (9.5)	51 048 (18.5)	77 008 (13.1)
Second quartile	13 171 (7.5)	13 143 (9.8)	51 734 (18.7)	78 048 (13.3)
Third quartile	17 397 (9.9)	18 209 (13.6)	41 900 (15.2)	77 506 (13.2)
Fourth quartile	28 152 (16.1)	24 965 (18.6)	23 252 (8.4)	76 369 (13.0)

^a^ Defined as age 18 years or younger.

^b^ Gender and race and ethnicity were generally self-identified but were occasionally reported by caseworkers.

**Figure 1.**
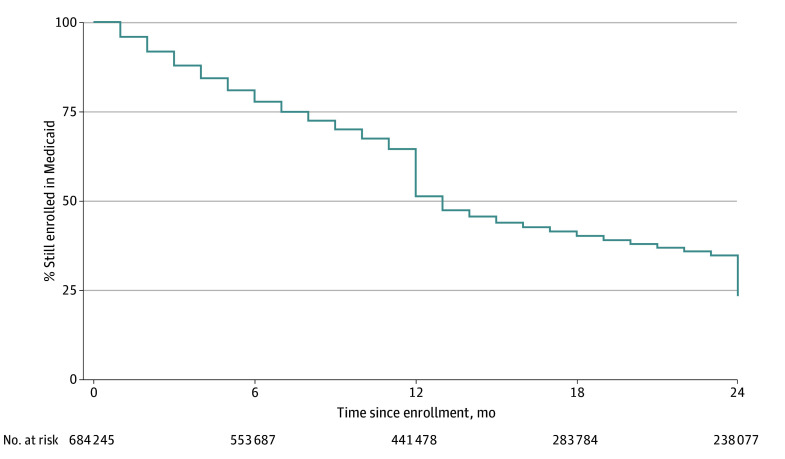
Duration of Medicaid Coverage The figure shows the percentage of people remaining continuously enrolled in Medicaid by months since the start of the Medicaid enrollment spell. Medicaid coverage dropped by 13 percentage points at the renewal deadline (end of month 12).

Figure 1 and Table 2 quantify the loss of Medicaid coverage before and after the renewal deadline for the full sample. Figure 1 presents a Kaplan-Meier curve capturing the duration of continuous Medicaid enrollment. Enrollment rates declined on a smooth path prior to the renewal deadline but then fell sharply, by 13 percentage points, at the deadline. Among people enrolled the month prior to the renewal deadline, 20% lost Medicaid coverage at the deadline.

**Table 2.** Loss of Medicaid Coverage Before and at the Renewal Deadline by Enrollee Characteristics

Characteristic	Coverage loss before the renewal deadline			Coverage loss at the renewal deadline		
	Total, No.	Lost coverage, No. (%)	*P* value^a^	Covered just prior to renewal deadline, No.	Lost coverage, No. (%)	*P* value^a^
No. of enrollment spells	684 245	242 767 (35.5)	NA	441 478	90 348 (20.5)	NA
Eligibility category						
Childless adult	203 205	88 392 (43.5)	<.001	114 813	40 110 (35.0)	<.001
Parent/caretaker	159 849	70 463 (44.1)		89 386	13 348 (15.0)	
Child	321 191	83 912 (26.1)		237 279	36 890 (15.6)	
Age, y						
<30	465 169	145 770 (31.3)	<.001	319 399	62 687 (19.6)	<.001
30-49	164 891	72 561 (44.0)		92 330	21 369 (23.1)	
≥50	54 185	24 436 (45.1)		29 749	6292 (21.2)	
Age of child, y						
<12	263 176	68 229 (25.9)	<.001	194 947	28 418 (14.6)	<.001
12-16	70 136	20 127 (28.7)		50 009	11 434 (22.9)	
Gender^b^						
Female	345 309	119 692 (34.7)	<.001	225 617	41 281 (18.3)	<.001
Male	338 936	123 075 (36.3)		215 861	49 067 (22.7)	
Education level						
<High school or missing	558 812	187 647 (33.6)	<.001	371 165	73 401 (19.8)	<.001
High school degree or equivalent	116 498	50 905 (43.7)		65 593	16 045 (24.5)	
>High school	8935	4215 (47.2)		4720	902 (19.1)	
Race and ethnicity^b^						
American Indian or Alaska Native, non-Hispanic	13 909	4769 (34.29)	<.001	9140	2095 (22.92)	<.001
Asian or Pacific Islander, non-Hispanic	24 171	7323 (30.3)		16 848	3077 (18.3)	
Black, non-Hispanic	133 677	49 898 (37.3)		83 779	19 359 (23.1)	
Hispanic	83 480	27 241 (32.6)		56 239	11 519 (20.5)	
Multiracial	27 364	9072 (33.2)		18 292	3542 (19.4)	
White, non-Hispanic	344 875	128 287 (37.2)		216 588	45 767 (21.1)	
Unknown	56 769	16 177 (28.5)		40 592	4989 (12.3)	
Urbanicity						
Metro area	488 905	174 400 (35.7)	<.001	314 505	66 967 (21.3)	<.001
Nonmetro area	160 009	57 573 (36.0)		102 436	19 925 (19.5)	
Missing	35 331	10 794 (30.6)		24 537	3456 (14.1)	
Income as % of federal poverty level at enrollment						
<25%	318 489	121 168 (38.0)	<.001	197 321	43 208 (21.9)	<.001
25%-75%	138 201	46 067 (33.3)		92 134	16 753 (18.2)	
>75%	215 276	71 416 (33.2)		143 860	29 689 (20.6)	
Missing	12 279	4116 (33.5)		8163	698 (8.6)	
US citizenship						
Noncitizen	13 881	4864 (35.0)	.27	9017	1616 (17.9)	<.001
Citizen	670 364	237 903 (35.5)		432 461	88 732 (20.5)	
Tribal membership						
Not a tribal member	667 398	237 135 (35.5)	<.001	430 263	87 915 (20.4)	.001
Tribal member	16 847	5632 (33.4)		11 215	2433 (21.7)	
Health care costs paid during first 6 mo of Medicaid enrollment						
None	324 137	186 853 (57.7)	<.001	137 284	57 461 (41.9)	<.001
First quartile	90 027	16 101 (17.9)		73 926	12 672 (17.1)	
Second quartile	90 034	13 132 (14.6)		76 902	8500 (11.1)	
Third quartile	90 020	13 209 (14.7)		76 811	6807 (8.9)	
Fourth quartile	90 027	13 472 (15.0)		76 555	4908 (6.4)	

Abbreviation: NA, not applicable.

^a^ χ^2^ Test of joint significance.

^b^ Gender and race and ethnicity were generally self-identified but were occasionally reported by caseworkers.

Table 2 also summarizes coverage loss by enrollee characteristics. There were statistically significant differences in the rate of coverage loss at the renewal deadline by enrollee eligibility category and by age. For example, while childless adults and parents had the same rate of Medicaid coverage loss before the deadline (44% for both groups), childless adults were more than twice as likely as parents to lose coverage at the deadline (35% vs 15%, respectively). Furthermore, only 15% of children younger than 12 years lost coverage at the redetermination deadline, compared with 23% of older children.

Rates of coverage loss at renewal also varied by the amount of health care costs Medicaid paid during the enrollee’s first 6 months of enrollment (Table 2). Among people enrolled just before the renewal deadline, 42% of those with no costs paid by Medicaid lost their coverage at the deadline, compared with 17% of people in the bottom quartile of health care costs and 6% of people in the top quartile. Indeed, for 64% of the people who lost coverage at the deadline, Medicaid had not paid any health care costs during their first 6 months of enrollment. In contrast, the differences in rates of coverage loss at renewal between income categories were very small, although statistically significant.

**Figure 2.**
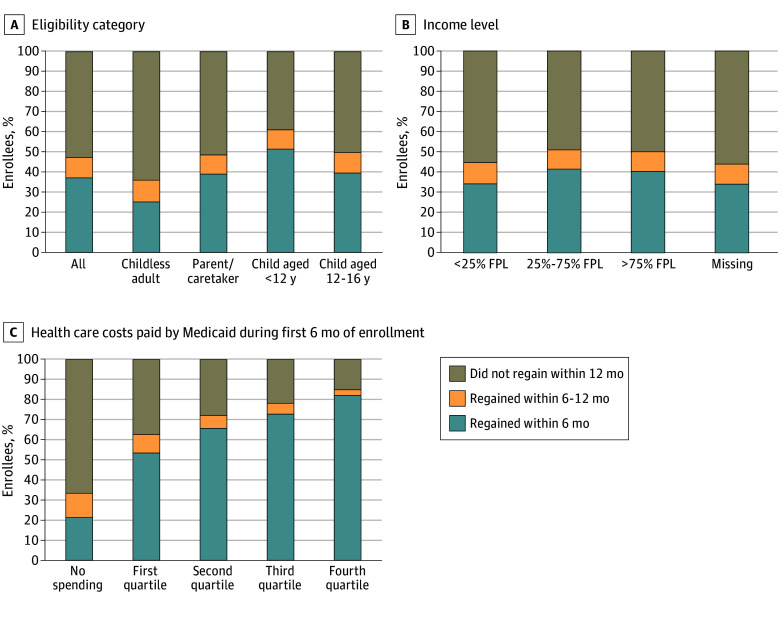
Duration of Coverage Gap for Enrollees Who Lost Coverage at Renewal Deadline by Enrollee Characteristics FPL indicates federal poverty level.

Figure 2 categorically depicts the duration of Medicaid coverage loss among those who lost coverage at the renewal deadline, overall and by enrollee characteristics (eFigure in Supplement 1 shows overall duration continuously). Overall, 37% of enrollees who lost coverage at the deadline regained Medicaid coverage within 6 months, and an additional 10% regained coverage between 6 and 12 months; the other 53% remained without Medicaid coverage for at least 12 months, as seen in Figure 2A. Figure 2A also depicts variations in the duration of coverage loss by age and eligibility category. While 64% of childless adults who lost Medicaid at the deadline remained without Medicaid for at least 12 months, children younger than 12 years often reenrolled quickly, with 51% regaining coverage within 6 months. Figures 2B and C show the duration of Medicaid coverage loss by income category and health care costs during the first 6 months of Medicaid enrollment, respectively. There is no clear gradient in duration of coverage loss by income, but there is a clear negative association between duration of coverage loss and past health care costs. For example, more than 82% of people with health care costs in the top quartile regained coverage within 6 months, compared with only 21% of enrollees who had no health care costs.

Table 3 summarizes the factors associated with losing Medicaid at the renewal deadline among people who had Medicaid coverage just before the deadline. The results of the first model show that factors associated with eligibility for and use of Medicaid services—eligibility category, household income at enrollment, and health care costs during the first 6 months of enrollment—are jointly associated with risk of losing coverage at the deadline. In the adjusted data, children and parents were 18.9 (95% CI, −19.2 to −18.6) and 17.1 (95% CI, −17.5 to −16.8) percentage points less likely, respectively, to lose their coverage at the deadline than childless adults (*P* < .001 for both). Income was also associated with coverage loss; people with a household income more than 75% of FPL at the time of enrollment were 4.9 (95% CI, 4.6-5.2) percentage points more likely than those with an income less than 25% of FPL to lose coverage at the deadline (*P* < .001). Finally, people with higher health care costs paid by Medicaid had a lower risk of losing coverage at the deadline, with the risk of coverage loss declining monotonically for each quartile of health care costs. People with costs in the highest quartile were 33.8 (95% CI, −34.1 to −33.5) percentage points less likely to lose coverage at the deadline than people with no health care costs (*P* < .001).

**Table 3.** Factors Associated With Losing Medicaid Coverage at Renewal Deadline by Logistic Regression Model^a^

Variable	Model 1: factors associated with eligibility for and use of Medicaid services		Model 2: factors from model 1 plus demographic characteristics	
	Average marginal effect (SE)	*P* value^b^	Average marginal effect (SE)	*P* value^b^
Eligibility category				
Parent/caretaker	−0.171 (0.002)	<.001	−0.184 (0.002)	<.001
Child	−0.189 (0.002)		−0.212 (0.002)	
Income as % of federal poverty level at enrollment				
25%-75%	0.007 (0.001)		0.008 (0.001)	
>75%	0.049 (0.001)		0.048 (0.001)	
Missing	−0.109 (0.003)		−0.102 (0.003)	
Health care costs during first 6 mo of Medicaid enrollment				
First quartile	−0.207 (0.002)		−0.205 (0.002)	
Second quartile	−0.271 (0.002)		−0.269 (0.002)	
Third quartile	−0.302 (0.002)		−0.300 (0.002)	
Fourth quartile	−0.338 (0.002)		−0.336 (0.002)	
Age group, y	NA	NA		<.001
30-49			−0.022 (0.002)	
≥50			−0.071 (0.002)	
Female gender			0.005 (0.001)	
Education level				
High school degree or equivalent			0.004 (0.002)	
>High school degree			−0.001 (0.005)	
Race and ethnicity				
American Indian or Alaska Native, non-Hispanic			0.024 (0.006)	
Asian or Pacific Islander, non-Hispanic			−0.043 (0.003)	
Black, non-Hispanic			−0.003 (0.002)	
Hispanic			0.015 (0.002)	
Multiracial			0.003 (0.003)	
Unknown			−0.047 (0.002)	
Urbanicity				
Nonmetro			−0.001 (0.001)	
Missing			−0.069 (0.002)	
US citizen			−0.002 (0.004)	
Tribal member			0.010 (0.006)	
Total No. of enrollment spells	441 478	NA	441 478	NA

Abbreviation: NA, not applicable.

^a^ The table reports the average marginal effects from logistic regression analyses of the probability of coverage loss during the Medicaid renewal process. Omitted categories are as follows: people eligible for Medicaid in the childless adult eligibility category, no health care spending during the first 6 months of coverage, income less than 25% of the federal poverty level, age younger than 30 years, male gender, less than high school or missing education, non-Hispanic White race, location of residence in a metro area, not a US citizen, and not a tribal member.

^b^ *F* test of joint significance.

The second model in Table 3 tests whether personal characteristics were associated with coverage loss after adjustment for factors associated with eligibility for and use of Medicaid services. Age, gender, race and ethnicity, education level, and metro area residence remained associated with the risk of losing Medicaid at the renewal deadline after adjustment for factors associated with eligibility for and use of Medicaid services. In the adjusted results, female enrollees were 0.5 (95% CI, 0.3-0.8) percentage points more likely to lose Medicaid at the deadline than male enrollees (*P* < .001). Compared with rates of coverage loss among non-Hispanic White enrollees, rates of loss at the deadline were 2.4 (95% CI, 1.2-3.7) percentage points higher among American Indian or Alaska Native enrollees (*P* < .001), 4.3 (95% CI, −4.8 to −3.8) percentage points lower among Asian or Pacific Islander enrollees (*P* < .001), not significantly different among Black enrollees, 1.5 (95% CI, 1.2-1.9) percentage points higher among Hispanic enrollees (*P* < .001), not significantly different among multiracial enrollees, and 4.7 (95% CI, −5.1 to −4.3) percentage points lower among those with unknown race (*P* < .001).

## Discussion

In this study of Medicaid beneficiaries with a renewal deadline 12 months after enrollment, 1 in 5 enrollees lost coverage at the renewal deadline. Among this group, 37% regained coverage within 6 months, whereas 53% remained without Medicaid coverage for at least 12 months. These lapses include both potentially avoidable coverage losses due to administrative burdens and coverage losses due to ineligibility.

These data describe which groups most frequently lose access to Medicaid at the renewal deadline and regain Medicaid coverage in the months after the deadline. When people in households with very low incomes (FPL <25%) lost Medicaid coverage at renewal, they did not regain coverage faster than their counterparts with higher income. Furthermore, the risk of Medicaid coverage loss varied by demographic factors even after adjustment for factors relevant to eligibility for and use of Medicaid services, with female enrollees, Hispanic enrollees, and Native American or Alaska Native enrollees showing elevated rates of coverage loss at renewal.

People with considerable health care needs may be more motivated to complete renewal processes, and the individuals or organizations providing their health care may be motivated to assist them. Past use of health care paid by Medicaid was strongly associated with loss of Medicaid coverage at renewal; in adjusted models, people in the highest quartile of health care use were 34 percentage points less likely to lose coverage at redetermination than people who used no health care. If people with no Medicaid-covered costs were removed from the sample, the risk of Medicaid loss at renewal would decline by half, to only 11%. In the present sample, more than half of enrollment spells had no Medicaid-paid health care costs during the first 6 months. During the COVID-19 public health emergency, when people may have been unaware that their Medicaid coverage had been extended,^30^ the proportion of people who rarely used their coverage may have been even higher.

In the present data, children younger than 12 years were the eligibility group most likely to regain Medicaid coverage quickly if they lost it, with half regaining coverage within 6 months. The consequences of short-term disruptions in Medicaid coverage include interrupted access to needed care^31,32^ and administrative costs for the Medicaid program of up to $500 each time an enrollee loses and regains Medicaid.^33,34^ If a rapid return to Medicaid coverage suggests that the enrollee lost coverage due to difficulties with the renewal process rather than because of ineligibility, then administrative barriers may account for a larger share of coverage losses among children than among other groups. Future research should consider the benefits and costs of making renewal requirements less frequent and how long to guarantee continuous coverage. The 2023 Consolidated Appropriations Act requires states to provide 12 months of continuous enrollment for children beginning in January 2024.^27^

### Strengths and Limitations

This analysis has several strengths. Importantly, we were able to isolate individuals who had Medicaid coverage just before the renewal deadline. Thus, the association between Medicaid-covered costs and the likelihood of losing coverage at renewal cannot be attributed to reverse causality (ie, people had no health care charges paid by Medicaid because they lost Medicaid coverage). The analysis also has important limitations. The sample does not include people who were originally eligible for Medicaid due to pregnancy or disability, and the effects of renewal requirements may differ for these groups. Furthermore, we cannot observe whether an enrollee took action to renew their coverage, including late renewals (which, if successful, would result in no loss of coverage). The data reflect the experiences of all beneficiaries, including those whose renewal was processed automatically (ex parte). Wisconsin uses ex parte renewals for less than half of its Medicaid population.^35^ Because ex parte renewal processes can increase rates of renewal,^36,37,38,39^ the results may not reflect the experience of states that certify eligibility of most enrollees on an ex parte basis. Finally, Wisconsin operates a joint Children’s Health Insurance Program–Medicaid program; churn between these programs may be relevant to beneficiaries in other states.

## Conclusions

Results of this cohort study highlight the strengths and limitations of current Medicaid renewal processes. Ostensibly, the goal of renewal requirements is to limit program receipt to those who qualify—in the focal sample, enrollees with sufficiently low income. Therefore, it may be surprising that people with very low baseline income (<25% of FPL) were not more successful in renewing their coverage than those with higher incomes. Future research should examine the extent to which nonrenewal reflects genuine changes in Medicaid eligibility vs logistical challenges such as address changes or gaps in internet or telephone access.^40,41^ Furthermore, the findings raise questions about why gender and race and ethnicity correlate with the risk of coverage loss at renewal. Future work should identify methods to refine renewal processes that ensure equitable access to Medicaid coverage among eligible people.

## Notes

Supplement 1. **eFigure.** Duration of time disenrolled from Medicaid coverage, among beneficiaries who lost coverage at the renewal date: a Kaplan-Meier analysis

Supplement 2. Data Sharing Statement
