# Supplementary material for: Loss of Medicaid Coverage During the Renewal Process
Source: JAMA Health Forum. 2024 May 3;5(5):e240839. doi: 10.1001/jamahealthforum.2024.0839 (PMC11069080; doi:10.1001/jamahealthforum.2024.0839)
Supplement: Supplement 1. — eFigure. Duration of time disenrolled from Medicaid coverage, among beneficiaries who lost coverage at the renewal date: a Kaplan-Meier analysis [file jamahealthforum-e240839-s001.pdf]

Supplemental Online Content

Dague L, Myerson R. Loss of Medicaid coverage during the renewal process. *JAMA Health Forum*. 2024;5(5):e240839. doi:10.1001/jamahealthforum.2024.0839

eFigure. Duration of time disenrolled from Medicaid coverage, among beneficiaries who lost coverage at the renewal date: a Kaplan-Meier analysis

This supplemental material has been provided by the authors to give readers additional information about their work.

© 2024 Dague L and Myerson R. *JAMA Health Forum.*

eFigure. Duration of time disenrolled from Medicaid coverage, among beneficiaries who lost coverage at the renewal date: a Kaplan-Meier analysis

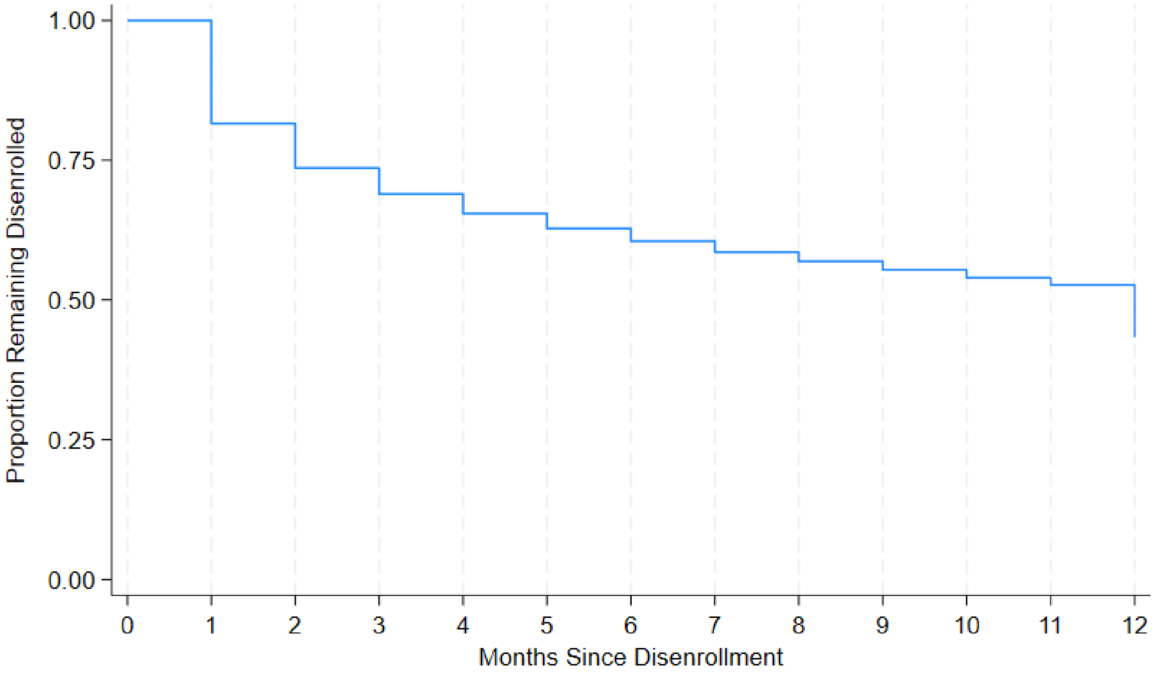

Source: Authors' calculations using Wisconsin administrative enrollment data

© 2024 Dague L and Myerson R. *JAMA Health Forum.*
