# Supplementary material for: Loss of Medicaid Coverage During the Renewal Process
Source: JAMA Health Forum. 2024 May 3;5(5):e240839. doi: 10.1001/jamahealthforum.2024.0839 (PMC11069080; doi:10.1001/jamahealthforum.2024.0839)
Supplement: Supplement 2. — Data Sharing Statement [file jamahealthforum-e240839-s002.pdf]

Data Sharing Statement

Dague and Myerson. Loss of Medicaid Coverage During the Renewal Process. *JAMA Health Forum*. Published May 03, 2024. doi:10.1001/jamahealthforum.2024.0839

Data
Data available: No

Additional Information
Explanation for why data not available: Data are not publicly available and require an application and data use agreement with Wisconsin DHS.
